# Analysis of marginal bone loss and implant stability quotient by resonance frequency analysis in different osteointegrated implant systems. Randomized prospective clinical trial

**DOI:** 10.4317/medoral.22742

**Published:** 2019-03

**Authors:** Rosa-María Díaz-Sánchez, José-María Delgado-Muñoz, María-Ángeles Serrera-Figallo, María-Isabel González-Martín, Daniel Torres-Lagares, José-Luis Gutiérrez-Pérez

**Affiliations:** 1 DDS, Department of Stomatology, Faculty of Dentistry, University of Seville, Spain; 2 DDS, Ph.D, Associate Professor, Department of Stomatology, Faculty of Dentistry, University of Seville, Spain; 3 DDS, Ph.D, Head Professor, Department of Stomatology, Faculty of Dentistry, University of Seville, Spain

## Abstract

**Background:**

The aim of the present prospective clinical study is to compare the stability of the implant-bone interface by the ISQ quotient and marginal bone loss (MBL) rate during one year of follow-up in four system implants with the same surface and different design.

**Material and Methods:**

Prospective randomized clinical trial of 21 patients in which four implant systems with the same surface and different design were placed. Patients were treated by the same operator following a similar surgical protocol with submerged technique. The second surgery to perform the prosthesis was performed at 3 months. All patients went to their review at 6 months and a year. A periapical radiograph for crestal bone analysis and an Implant stability quotient by resonance frequency analysis (ISQ) analysis were taken at baseline and the reviews.

**Results:**

No statistically significant differences were found in the Implant stability quotient by resonance frequency analysis and Marginal Bone Loss in the four types of implants. The ISQ increased from the moment of insertion of the implant until the revision to the year, showing an increase of the stability implant, being this increasing less between the 6 months and the year.

**Conclusions:**

Differences in the design of the four implants tested in this study did not show statistically significant differences in any of the variables studied, so the implant design does not influence implant stability and marginal bone loss in the first year after placement.

** Key words:**Marginal bone loss, implant stability quotient, dental implant, clinical trial.

## Introduction

The replacement of missing teeth with implant-supported restorations has become an accepted treatment modality for partially and totally edentulous patients. According to clinical studies, the long-term survival of dental implants has exceeded 96%, which makes it a more accepted and sought treatment by our patients. A stable and aesthetic implant restoration can be achieved only through careful consideration of the biological principles of peri-implant hard and soft tissue healing, as well as the selection of an appropriate implant type and position (1-5).

Marginal bone loss originates from a combination of mechanical and biological factors, and factors hypothesized to be associated with marginal bone loss include the surgical trauma to the periosteum and bone, size of the microgap between the implant and the abutment, bacterial colonization of the implant sulcus, the biological width, and the biomechanical factors related to loading (1,7).

Besides the biological factors, modifications have been made to the design to improve the biological response of tissues to prevent or reduce marginal bone loss (MBL), an optimal stress loading distribution, implant primary stability and better periodontal adaptation (11).

Nowadays, we can found more than 100 implant systems in different designs. Numerous papers assure that microthreads in the crestal portion can reduce marginal bone loss (MBL) around implants (1,5-8). Clinical studies have shown that rough surfaced implants with microthreads at the neck can maintain the marginal bone level during the healing period and cause significantly less MBL under long-term functional loading (1,9). Microthreads location is important in reducing MBL, the amount of MBL around implants with a roughened neck is less than with a polished neck because a higher compression and less shear stress al the crestal bone is produce and reduce the marginal bone resorption(1,9,10). However, other articles assure that the polished neck produces a better periodontal tissues adaptation (11), have the same results than a roughened neck (12) or that there is no significant difference between implants with macrothreads and microthreads in terms of MBL after loading (13-15).

The implant design also influences on the stability of the implant, after the surgery and during the osteointegration and loading time. Implant stability quotient by resonance frequency analysis (ISQ, Osstell) can provide clinically relevant information on implant stability immediately after insertion and at selected time points thereafter. It evaluates implant stability as a function of the stiffness of implant-bone interface and it is influenced by factors such as bone density, jaw healing time and exposed implant height above the alveolar crest (3). The ISQ measurement was found to be reproducible irrespectively of the instrument positioning. ISQ values were affected by the bone structure and implant length, therefore some authors have concluded that no predictive values can be attributed to implant stability (16-18).

The aim of the present prospective clinical study is to compare the stability of the implant-bone interface by the ISQ quotient and marginal bone loss (MBL) rate during one year of follow-up in four system implants with the same surface and different design.

## Material and Methods

-Study Desing

A randomized, prospective, split-mouth clinical trial was conducted among 20 consecutively patients from the School Dentistry at the University of Sevilla, in which four different implant systems of TiPurePlus BEGO Implant Systems ® were placed with one year follow up.

Patients with implant needs were cited in the master of Oral Surgery at the University of Seville. Those patients who met the inclusion criteria for the study were selected. The implants were placed following a randomized distribution.

The study protocol was approved by the Ethics Committee of the University of Sevilla. Prior to participation, the purpose and procedures were fully explained to all patients and all participants gave written informed consent in accordance with Helsinki declaration (2002 version, www.wma.net/e/policy/b3.htm). The study was designed, conducted, analysed and reported according to guidelines for Good Clinical Practice.

The four systems of implants placed in our 20 patients are the four standard models that have the BEGO Implant Systems ® company, excluding in the present study the Line Mini Semados TiPurePlus.

The BEGO Semados ® S implants are self-tapping implants with 0.8mm cylindrical polished mechanized shoulder for binding to the mucosal tissue with less irritation. The apex of the implant is rounded to protect anatomical structures.

The BEGO Semados RI ® implants are self-tapping implants with 0.5mm tapered polished mechanized shoulder for insertion of soft tissue. This implant has a polished mechanized shoulder of lower extension to incorporate microgrooves in the neck region to improve the load transfer to the bone crest.

The BEGO Semados Rs ® has a mechanized shoulder implant whose surface has a micro roughness close to that of tooth enamel (Ra ≈ 0.4) and microthreads next to the mechanized shoulder. It has a chamfer on the shoulder of the implant, which leads to minimization of mechanical stress to the implant during the masticatory load.

The BEGO Semados RSX ® has a shoulder implant treatment TiPurePlus without polished mechanized shoulder, it has microthreads in the shoulder. It also has a chamfer on the shoulder of the implant, which leads to minimization of mechanical stress to the implant during the masticatory load.

The microthreads in the implant head that were incorporated in the previous version, BEGO Semados ® RI, have been optimized bionically.

The implants will be placed in the maxilla or mandible; in no case there will be comparison between implants placed in both jaws, since the quality and bone physiology is different, causing a bias in the study. In addition, bone quality will be collected to apply the variable to the data.

All implants have TiPurePlus surface, which means that they are treated with aluminum oxide sandblasting and acid etching. Diameters included in the clinical trial are 3.8; 4.1; 4.5 and 5.5; and length included are 9mm, 10mm, 11.5mm and 13mm.

All implants are tapered internal connection 45 Internal Hex, in which is equivalent abutments system, so it is possible compare the four designs of this company purely.

At the pre-screening visit the Medical and Dental histories were taken and the screening was carried out base on the inclusion and exclusion criteria. Inclusion criteria were: subjects older than 18 years, male or female, good general health, smokers of less than 10 cigarettes per day, patients with an edentulous maxillar or mandible which needs screwed hybrid prosthesis, signed informed consent before study initiation.

-Surgery

A preoperative antibiotic medication was not administrated in our patiens. Before surgery they performed 0.12% chlorhexidine rinses to reduce the bacterial load. Usual aseptic measures for implant surgery were taken.

Patients will be performed under local anesthesia with 0.36ml of Lidocaine Hydrochloride and Epinephrine 1:80,000.

The four implant systems were being placed in the same surgical procedure, following a standard protocol for implant placement. BEGO Implant Systems ® drilling kit were used for drillling the bone accroding to each type of implant. Implant placement was carried out with copious irrigation with sterile solution to avoid overheating the surgical site.

The implants were placed subcrestally, after insertion of the implants, the cover screw was placed until the osteointegration time.

-Healing Abutment Placement

After this period of three months the implants will be uncovered and a healing abutment will be placed to form the soft tissue. At this time, it will be held the study measurements again, periapical radiographs will be taken and the ISQ index measured.

-Prosthesis Placement

The implants were charged after second surgery, after 3 months of placement.

The hybrid prosthesis was fitted with screw fixation, cemented in no case. With the placement of the screwed prosthesis, we could remove the load for testing the bone stability after six months and a year of implant placement.

-Study parameters

Three evaluation visits were performanced, according to each phase of the treatment, during the study after the surgery for an oral examination and to determine the bone stability with a periapical radiography and the ISQ index.

• A measurement of the frequency index magnetic resonance in ISQ units was helded with the Ostell ISQ®. ISQ (implant stability coefficient) is a measurement scale for use with the RFA (resonance frequency analysis), method for determining the stability of an implant. It is a representation of the resonant frequencies (kHz), presented on a scale of 1-100 ISQ. ISQ was registered four times; immediately after implant surgery using the contact free, third generation device Osstell (Malmö, Sweden), at the 2nd stage surgery (uncovering). The sc were removed 6 months and 1 year after implant placement for ISQ measurement. Four measurements were carried out in each implant (buccal, mesial, distal and lingual or palatine), the average was found to obtain a single value for implant in each moment described.

• Periapical radiographs of the implants were taken with a paralleliser to determine the level of crestal bone. The MBL mesurements were done in the mesial and distal bone and were measured in millimeters from the implant shoulder.

## Results

A total sample of 20 patients completed the clinical trial. The mean age at the time of surgery was 64.5 ± 10,22 years. 38% of patients were smokers that reported the consummation of less than 10 cigarettes / day. 61% patients had a history of previous periodontal disease.

The patients have received 106 implants BEGO Semados ®: 35 RI, 27 S, 22 RSX and 22 RS (platform diameter 3.75mm in 45 implants, 4.1mm in 41 implants, 4.5mm in 16 implants and 5.5mm in 4 implants; implant length 8.5mm in 27 implant, 10mm in 56 implants, 11.5mm in 11 implants and 13mm in 12 implants) with TiPurePlus surface.

Once analysed the study groups, it was conducted the data processing depending on the different views held.

-ISQ

The ISQ measurements do not showed statistical significant differences at baseline between the implant groups and in the subsequent visits (*p*<0.005). The results obtained showed an increasing ISQ measurements throughout the visits. The tendency was similar in each implant group ( Table 1).

**Table 1:**
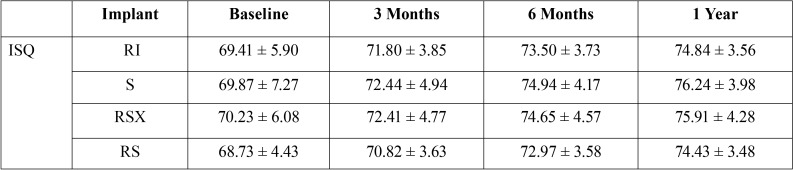
ISQ data in the group of study.

-Crestal Bone

The marginal bone loss (MBL) measurements do not showed statistical significant differences between the baseline and the data in the subsequent visits (*p*<0.005). The MBL was similar between after one year and no statistically significant differences were found between groups ( Table 2).

**Table 2:**
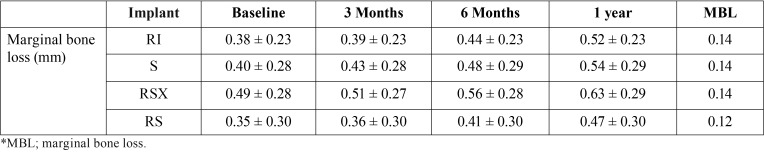
Marginal bone loss data in the group of study.

## Discussion

Primary stability depends on the bone quality, surgical technique, and implant design (19). Adequate primary stability is essential to achieve clinical success. In our study, four different implants of the same company have been studied with different design. No statistically significant differences were found between groups in each two parameters studied. However, the ISQ indexes increased from the first visit to the year, showing higher implant stability after one year. The increasing was gradual, being lower from the 6 months visit to the year.

Although there were no statistically significant differences, RSX implant achieved the highest ISQ quotient with a 70.23 ± 6.08, this fact could be explained by the micro-threats in the shoulder rather than the implant body, which is the same of the RS implant, which achieved the lowest quotient (68.73 ± 4.43). This fact is in accordance with the publication done by Abuhussein *et al.*, who assure that the macrodesing of the implants helps to achieve the primary stability (7), as well as the distribution load. Waechter *et al.* also published a clinical trial in May 2017 in which assure that there were no statistically differences between tapered and cylindrical implants, so they concluded that tapered and cylindrical implants have similar biological behaviour during the healing process, as we concluded after our clinical trial, in which the ISQ quotient do not have statistically significant differences (18).

Likewise, no statistically significant differences were found in crestal bone loss between the four implant systems from the baseline to the year visit. RS Bego Implant System was the one that less bone loss regardless that there were no statistically differences between groups. The MBL after one year was 0.12mm versus the 0.14 mm in the other groups. RS Bego Implant System has a polished neck of 0.5mm before the microthreads. However, some authors propose that the microtheads around the neck implant brings resistance to MBL during the fist phases of healing (5-7). Niu et al also published a meta-analysis in which assure that microthreads can reduce the MBL but also assure that the differences were small in the articles and there are no so much clinical trials published (1). Only five clinical trials were included in the systematic review. However, other articles propose that the polished neck stabilizes the periodontal tissues and this fact helps to reduce the crestal bone loss by external factors as the peri-implantitis. Sánchez-Siles *et al.* published a retrospective study in which concluded that implants with smooth polished necks suffered less bone loss and peri-implantitis during 10 years (11). The optimal results obtained in this study were on 2.5mm smooth neck implants in short, medium and long terms. Der Hartog *et al.* also published clinical trial in 2011 in which assure that implants with 1.5 smooth polished neck and rough neck with threads did not present significant statistically differences in the MBL (12). These results could be compared with results obtained in our study.

As we explained above, the implant in which less MBL was observed after one year was the RS Bego Implant although no statiscally differences were found. The neck design combines a polished shoulder of 0.5mm and microthreads. The combination seeks to stabilize the periodontal tissues and decrease the rate of peri-implantitis, properties attributed to the polished neck in the literature, as well as decrease the MBL by the microthreads. This innovative design reduces stress peaks in the crestal bone, so the maximum load is not accumulated in the at the insertion point, it is moved distally. This is achieved with an equitable distribution of forces, greatly reducing the risk of a non-physiological overload.

## Conclusions

No significant statistically differences between the four Bego Implants Systems were found in the Implant stability quotient by resonance frequency analysis (ISQ) and in the Marginal Bone Loss after a year follow-up.

Further research on the designs is needed to clarify the mechanism and the relationship between implant design and crestal bone loss and the stability.
